# Assessing the cost of acute stroke care in Ethiopian public tertiary hospitals: a multicenter study

**DOI:** 10.3389/fneur.2026.1664986

**Published:** 2026-03-27

**Authors:** Kemal Ali, Sheila Ouriques Martins, Biniyam Alemayehu Ayele, Girma Diltata, Zenawi Hagos Gufue, Yared Mamushet Yifru

**Affiliations:** 1 Neurology Unit, Department of Internal Medicine, Yekatit-12 Hospital Medical College, Addis Ababa, Ethiopia; 2 Stroke Unit, Universidade Federal do Rio Grande do Sul, Hospital de Clínicas de Porto Alegre, Porto Alegre, Rio Grande do Sul, Brazil; 3 Neurology Service, Hospital Moinhos de Vento, Porto Alegre, Rio Grande do Sul, Brazil; 4 World Stroke Organization, Geneva, Switzerland; 5 Department of Neurology, College of Health Sciences, Addis Ababa University, Addis Ababa, Ethiopia; 6 Global Brain Health Institute, University of California, San Francisco, San Francisco, CA, United States; 7 Department of Public Health, College of Medicine and Health Sciences, Adigrat University, Adigrat, Ethiopia; 8 Department of Epidemiology and Biostatistics, Arnold School of Public Health, University of South Carolina, Columbia, SC, United States

**Keywords:** acute stroke care, cost-of-illness, developing countries, economic burden, Ethiopia

## Abstract

**Background:**

The cost of acute stroke data is scarce in developing countries, especially Ethiopia, despite its significance for public health. This study aimed to assess the total cost of acute stroke care per patient per year among acute stroke patients admitted to tertiary hospitals in Addis Ababa, Ethiopia.

**Methods:**

An incidence-based, prospective, cross-sectional cost-of-illness study was conducted among 99 acute stroke survivors admitted to two specialized hospitals in Addis Ababa. A micro-costing technique was applied to pinpoint cost-generating elements and assign the proper unit costs for the two cost categories, direct and indirect. A multivariable generalized linear model was employed to identify predictors of acute-phase costs among stroke patients.

**Results:**

The median cost of acute stroke care per patient per year was $286, with direct and indirect costs of $193.77 and $74, respectively. Between-group cost differences were primarily driven by delayed hospital presentation (arrival >4.5 h after symptom onset; adjusted cost ratio [CR] 1.78), ICU admission (CR 1.56), older age (CR 1.27), at least one comorbid condition (CR 1.27), and rural residence (CR 1.40).

**Conclusion:**

The costs of acute stroke care in public tertiary hospitals in Ethiopia are very high, with over 30% of GDP per capita spent on acute stroke care. Understanding the cost of stroke in Ethiopia is critical for planning the implementation of acute care services, including stroke treatments, under universal health coverage, ensuring that all stroke patients benefit.

## Introduction

Approximately 85% of deaths from stroke occur in low- and middle-income countries (LMICs) (1). Age-and disability-adjusted life-year (DALYs) loss rates and stroke mortality rates are 3.5 to 3.8 times higher in low-income countries (LIC) than in middle- and high-income countries (1). African countries are experiencing epidemiological changes driven by socio-demographic and lifestyle changes (2). The burden of non-communicable diseases, including cardiovascular risk factors, is increasing. Thus, stroke is a major complication of cardiovascular risk factors, and its incidence appears to be rising in Africa and other LMICs (2). In the United Kingdom, the cost of providing acute and long-term care, combined with the estimated costs of lost productivity for stroke patients, exceeds £8 billion a year (3). In the United States, annual costs have reached $33.6 billion, of which $7.6 billion is directly attributable to hospitalizations, representing a major financial burden on health services (3).

Considering the economic burden of stroke in developed countries and the young age at which stroke occurs, particularly in sub-Saharan Africa (4), there is an urgent need to address the financial and healthcare challenges posed by stroke. Stroke is an expensive disease, and most stroke-related costs come from hospitalization (5). In the first year, approximately 30% of stroke costs are spent on acute inpatient care and 30% on inpatient rehabilitation. With the expected increase in stroke incidence associated with an aging population, it is important to find more efficient and cost-effective ways to provide hospital care for acute stroke in resource-limited settings (5).

A 2014 systematic review sponsored by the World Stroke Organization found that stroke‑related costs vary widely across low‑ and middle‑income countries, largely due to differences in healthcare system structures. The two main drivers of cost were stroke severity and duration of hospitalization. The highest reported direct medical expenditure for a stroke episode was US$8,424 in Nigeria, while the lowest was US$416 in Senegal. The longest average hospital stay, 20 days, was observed in China. The review also noted that the available evidence was limited by a lack of standardized research methods on the topic (4).

There are very few studies in Africa on the cost of stroke treatment, with one in Togo estimating direct costs per capita at €936 for just 17 days, which was about 170 times the average annual health expenditure of Togolese (6). In Ethiopia, no previous studies have examined the costs of stroke care, neither acute hospitalization nor chronic rehabilitation costs. To enact better financial policies for prevention, hospital treatment, outpatient rehabilitation programs, and social services, policymakers need information on how much is spent treating acute stroke patients. Hence, this study aimed to assess the total cost of acute stroke care per patient per year among acute stroke patients admitted to tertiary hospitals in Addis Ababa, Ethiopia.

## Methods and materials

### Study area and period

The study was conducted in the medical wards and intensive care units of two public teaching and referral hospitals, Tikur Anbessa Specialized Hospital (TASH) and Yekatit-12 Hospital Medical College, in Addis Ababa, Ethiopia. Currently caring for more than 400,000 patients annually, TASH is the largest referral teaching hospital in the nation. The hospital has 13 neurologists, residents, nurses, and other supportive staff. Yekatit 12 Hospital, with its medical college, is currently serving more than 500,000 patients per year and it has facilities for a range of services, including preventive and rehabilitative activities.

There was no acute stroke unit or protocol in place at either hospital during the study period. Both hospitals have an emergency room, medical wards, and adult intensive care units. The hospitals have echocardiography and carotid Doppler ultrasound. Some laboratory investigations (anticardiolipin antibody and lupus anticoagulant) and thrombectomy are lacking and patients were referred to private centers. The TASH has both computed tomography scan and magnetic resonance imaging but is usually not functional, though it used to work. Unless the patient has health insurance, all patients are expected to pay for all interventions or services they are receiving. Data were collected from December 1, 2020 to November 30, 2021.

### Study design and sampling procedures

A multicenter, incidence-based, prospective, cross-sectional cost-of-illness study was conducted among 99 adult acute stroke patients using a consecutive sampling technique in the two hospitals.

### Eligibility criteria

The study comprised adult patients (≥18 years old) whose diagnosis of stroke events (primary and recurrent) was verified by a computerized tomography (CT) scan and/or magnetic resonance imaging (MRI).

Patients were excluded from the study if they had at least one of the following conditions that developed during their hospital stay: cancer, fatal renal, hepatic, or patients with severe strokes that need mechanical ventilation, disabling and progressive neurological diseases like multiple sclerosis and Parkinson’s disease, and dementia.

### Data collection tools and procedures

A pretested, structured, interviewer-administered questionnaire adapted from previous cost-of-stroke studies was used to collect data from patients and their caregivers. Six experienced nurses conducted the interviews at the two hospitals. The questionnaire captured sociodemographic characteristics (age, sex, educational status, occupational status, and income level) and clinical characteristics, including co-morbidities and stroke type (ischemic or hemorrhagic; first-ever or recurrent). Stroke severity at admission was assessed using the National Institutes of Health Stroke Scale (NIHSS).

The questionnaire also included an economic module that captured both direct and indirect costs related to the index stroke. Cost data were recorded at three points: (1) during the index hospital admission, (2) at discharge, and (3) during all stroke-related health-care contacts occurring after discharge. We defined “subsequent visits” as any stroke-related contact with a health facility within 12 months of the index admission, including neurology or medical outpatient appointments, emergency department visits, and any stroke-related re-admissions at the study hospitals or other health facilities. Data on resource use during hospitalization and at subsequent visits were collected prospectively from patients and caregivers using the same structured questionnaire.

### Cost of analysis

Cost information was collected prospectively from the time of the index hospitalization through 12 months of follow-up for each patient. We applied an incidence-based micro-costing approach from the perspective of patients and their families. Costs are reported in 2021 US dollars, using the average exchange rate during the study period (44.30 Ethiopian birr per 1 USD). The economic questionnaire was designed to capture only expenditures that patients or caregivers attributed to the stroke episode. For each resource item, we recorded the quantity of use at the individual level (e.g., number of hospital days, number and type of investigations, and quantities of medications) and multiplied these quantities by the corresponding unit costs. Total direct cost per patient was then calculated as the sum of all items.

Direct costs were defined as all medical and non-medical payments made by the patient or family to obtain care for the index stroke. In the study hospitals, unless the patient has health insurance, all services are paid out-of-pocket; therefore, the recorded direct costs predominantly represent out-of-pocket expenditures. We did not adjust costs for any subsequent insurance reimbursements. Direct costs were grouped into five categories:

1. *Transportation costs*: All payments for travel of the patient and at least one accompanying caregiver between home and any health facility for stroke care, using ambulance services, taxis, buses, or private vehicles, at the time of the index admission and for subsequent stroke-related visits during follow-up.
2. *Investigation costs*: Payments for all diagnostic tests related to the stroke, including routine laboratory tests (complete blood count, serum electrolytes, renal and liver function tests, lipid profile, coagulation profile, blood glucose tests, inflammatory markers, viral markers, and urinalysis); neuroimaging (non-contrast brain CT and/or brain MRI); and other investigations such as electrocardiogram (ECG), echocardiography, chest X-ray, and carotid Doppler ultrasound.
3. *Medication costs*: Expenditures on all prescribed drugs and related medical supplies used for stroke management during the index hospital stay and at stroke-related outpatient visits within 12 months after stroke (e.g., antiplatelet and anticoagulant agents, antihypertensives, statins, antidiabetic drugs, antibiotics, analgesics, and intravenous fluids).
4. *Inpatient service costs*: Payments for hospital bed-day charges in the general medical ward or intensive care unit (ICU). These bed-day tariffs include routine nursing care and ward consumables bundled into a single daily charge. Inpatient service cost per patient was calculated as the bed-day charge multiplied by the total length of stay across all admissions.
5. *Other direct costs*: Additional stroke-related expenditures not captured in the categories above, primarily payments for consultations, investigations, and treatments at other health facilities (such as primary hospitals, health centres, and private clinics) for the same illness episode, both before referral to the study hospitals and during the follow-up period.

For services delivered within the two study hospitals, unit costs for inpatient bed-days, investigations, and medications were obtained from hospital billing and finance offices and from hospital pharmacy price lists. For services obtained outside the study hospitals (private diagnostic centres, laboratories, imaging facilities, and community pharmacies), we used patient receipts when available. When receipts or exact amounts could not be produced, we collected current price lists from diagnostic centres, laboratories, and pharmacies in Addis Ababa and calculated the mean unit price for each investigation and medication. These mean prices were then applied as unit costs. If the patient or caregiver was unable to recall the precise amount paid for a given service, we assigned the corresponding average unit cost.

Direct cost estimates covered the entire 12-month period after the index stroke, not only the initial admission. Thus, in addition to costs accrued during the index hospitalization, we included post-discharge expenditures for stroke-related outpatient visits, emergency department attendances, additional investigations, medications, transportation for follow-up, and any stroke-related re-admissions within 1 year.

Indirect costs reflected productivity losses due to stroke for both patients and their main caregivers over the same 12-month period. We used the human capital approach: for employed patients and caregivers, each day of work lost because of hospitalization, outpatient visits, or caregiving responsibilities was valued at the self-reported average daily income from their main occupation. For unpaid caregivers, we used a replacement cost approach to value time spent providing informal care. Total indirect cost per patient was calculated as the sum of productivity losses for the patient and caregiver. When a patient or caregiver could not recall the precise income loss, we used the average value observed among other respondents. Supplementary Table 1 details investigation costs; Supplementary Table 2 presents individual-level cost components.

### Statistical analysis

Data were collected using a structured, interviewer-administered paper questionnaire. The completed paper forms were entered into Microsoft Excel spreadsheets and then exported to SAS software, version 9.4 (TS1M9; SAS Institute Inc., Cary, NC, USA) for data management and analysis. All datasets and variables were checked and cleaned prior to analysis, including verification of ranges, internal consistency, and patterns of missingness.

Because the cost variables were right-skewed, continuous variables were summarized by median and inter-quartile range (IQR), and categorical variables as frequencies and percentages. We used the Mann–Whitney *U* test for comparing continuous or ordinal variables between two groups, and the Kruskal–Wallis test for more than two groups.

To identify factors associated with total acute stroke care cost per patient, we modeled total cost using a generalized linear model with a gamma distribution and log link, which is appropriate for strictly positive, right-skewed, and potentially heteroskedastic cost data. Candidate predictors were selected based on clinical relevance and bivariable analyses; variables with a *p*-value <0.10 in bivariable analyses were entered into the multivariable model, and statistical significance at *p* < 0.05 was used to inform the final model specification.

Model diagnostics based on Pearson and deviance residuals indicated adequate fit, with no apparent systematic patterns and most residuals falling within ±1.5 standardized units. Residual heteroskedasticity was explored using a Breusch–Pagan–type test applied to the deviance residuals. Multicollinearity among covariates was evaluated using variance inflation factors (VIFs). Overall model performance was summarized using deviance statistics and a Cox–Snell–type pseudo-*R*^2^, which provides a descriptive measure of relative model fit rather than a direct analogue of the *R*^2^ from linear regression.

Deterministic sensitivity analyses were conducted to assess the robustness of cost estimates. One-way analyses varied key cost drivers within empirically observed ranges, and scenario analyses evaluated low-, base-, and high-cost assumptions, including a conservative scenario accounting for the exclusion of mechanically ventilated patients. Costs were also examined under alternative currency conversion assumptions and expressed as a proportion of national GDP per capita.

## Results

### Socio-demographic characteristics of the patients

Out of a total of 118 participants, 99 patients with complete data were included in the study, giving a response rate of 84%. The largest age group was 61–80 years, comprising 43% patients, followed by 31% patients aged 41–60 years. More than half of the patients were male (57.6%). Most patients resided in urban areas (77.8%), and the most common occupation category was private employment (42.4%), followed by unemployed (30.3%) and retired (16.2%). Among the variables examined, marital status (*p* = 0.0099) and area of residence (*p* = 0.0008) showed statistically significant differences in total cost per patient, whereas age group, sex, educational status, occupation, and religion were not significantly associated with total cost (Table 1).

**Table 1:** Socio-demographic characteristics of acute stroke patients admitted to Tikur Anbessa Specialized Hospital and Yekatit-12 Hospital Medical College, Addis Ababa, 2021 (*n* = 99).

Baseline characteristic	Category	*n* (%)	*p*-value^*^
Age group (years)	18–40	14 (14.1)	0.5495^†^
	41–60	31 (31.3)	
	61–80	43 (43.4)	
	>80	11 (11.1)	
Sex	Male	57 (57.6)	0.1642^‡^
	Female	42 (42.4)	
Marital status	Married	64 (64.7)	0.0099^†^
	Widowed	17 (17.2)	
	Divorced	7 (7.1)	
	Single	7 (7.1)	
	Separated	4 (4.0)	
Educational status	Unable to read and write	31 (31.3)	0.6613^†^
	Able to read and write	20 (20.2)	
	Primary school	12 (12.1)	
	Secondary school	23 (23.2)	
	College and above	13 (13.1)	
Residence	Urban	77 (77.8)	0.0008^‡^
	Rural	22 (22.2)	
Occupational status	Private	42 (42.4)	0.5195^†^
	Unemployed	30 (30.3)	
	Retired	16 (16.2)	
	Government employee	10 (10.1)	
	Student	1 (1.0)	
Religion	Orthodox Christian	76 (76.8)	0.5550^†^
	Muslim	15 (15.2)	
	Protestant	7 (7.1)	
	Catholic	1 (1.0)	

*p-value for difference in total cost per patient between/among patient characteristics, based on non-parametric tests.

^†^Kruskal–Wallis test. ^‡^Wilcoxon rank-sum (Mann–Whitney) test.

### Clinical characteristics of the patients

The median length of hospital stay was 9 days (IQR 6–12). Only 14% patients arrived at the hospital within 4.5 h of symptom onset: 7% arrived within 3 h, 7% between 3 and 4.5 h, while the majority (85.9%) arrived after 4.5 h. Ischemic stroke was the predominant type, accounting for 67.7% patients, whereas 32.3% had hemorrhagic stroke. Previous stroke was reported in 9.1% patients, and 69.7% had at least one comorbid illness such as hypertension, diabetes mellitus, or dyslipidemia.

At admission, stroke severity based on NIHSS was: 26.3% minor (1–4), 46.5% moderate (5–15), 17.2% moderate-to-severe (16–20), and 10.1% severe (21–42). Non-parametric analysis of total cost per patient showed significant associations with onset-to-arrival time (*p* < 0.0001), stroke type (*p* = 0.002), presence of medical complications (*p* = 0.01), admission NIHSS category (*p* = 0.03), place of admission (general ward vs. ICU; *p* = 0.02), and presence of comorbid illness (*p* = 0.002). History of previous stroke was not significantly associated with total cost (*p* = 0.14) (Table 2).

**Table 2:** Clinical characteristics and their association with total cost of acute stroke care among acute stroke patients admitted to Tikur Anbessa Specialized Hospital and Yekatit-12 Hospital Medical College, Addis Ababa, 2021 (*n* = 99).

Clinical characteristic	*n* (%)	*p*-value*
Onset-to-arrival time (hours)		<0.0001^†^
<3	7 (7.1)	
3–4.5	7 (7.1)	
> 4.5	85 (85.9)	
Stroke type		0.0019^‡^
Ischemic stroke	67 (67.7)	
Hemorrhagic stroke	32 (32.3)	
History of previous stroke		0.1392^‡^
Yes	9 (9.1)	
No	90 (90.9)	
Medical complications		0.0146^†^
No complication	74 (74.8)	
Aspiration pneumonia	16 (16.2)	
Urinary tract infection	5 (5.1)	
Other complications	4 (4.0)	
Admission NIHSS score		0.0345^†^
Minor (1–4)	26 (26.3)	
Moderate (5–15)	46 (46.5)	
Moderate to severe (16–20)	17 (17.2)	
Severe (21–42)	10 (10.1)	
Place of admission		0.0185^‡^
General ward	95 (96.0)	
ICU	4 (4.0)	
Co-morbid illness		
Yes	69 (69.7)	0.0019^‡^
No	30 (30.3)	

*p-value for difference in total cost per patient between/among patient characteristics, based on non-parametric tests.

^†^Kruskal–Wallis test. ^‡^Wilcoxon rank-sum (Mann–Whitney) test.

ICU, intensive care unit; IQR, interquartile range; NIHSS, National Institutes of Health Stroke Scale.

### Direct, indirect, and total costs of acute stroke care

In this study, the total direct costs of acute stroke care included the sum of costs for transportation, laboratory tests and imaging modalities, medications, inpatient services, and other healthcare facility visits for the current illness before reaching the study hospitals. The minimum and maximum direct costs are $55.7 and $844.2, respectively. The median direct cost of acute stroke care was $193.77 per patient; likewise, the median cost of investigations was $76.26 per patient during their course of care in the hospital. The median cost of inpatient service was $50.16 and the median cost of medication was $23.98 per patient during their course of care in the hospital. Neuroimaging and laboratory costs drove the majority of direct acute stroke care costs.

Ninety-seven (98%) patients underwent non-contrast brain CT scan imaging and two patients had MRIs from the beginning. The median cost of a non-contrast brain CT scan was $45.13, and 11 patients (11%) required additional brain MRI to settle their diagnosis. The median cost of a brain MRI was $90.25. The total median cost of brain imaging, including both CT scans and MRIs, was $45.13. The median laboratory cost was $31.7, which included a complete blood cell count, coagulation profile, liver function test, renal function test, serum electrolytes, erythrocyte sedimentation rate, c-reactive protein, viral markers, urine analysis, fasting blood sugar, random blood sugar, lipid profile, and cardiac biomarkers. The median cost of other investigations (ECG, echocardiography, chest X-ray, and carotid Doppler) was $2.71.

**Figure 1:**
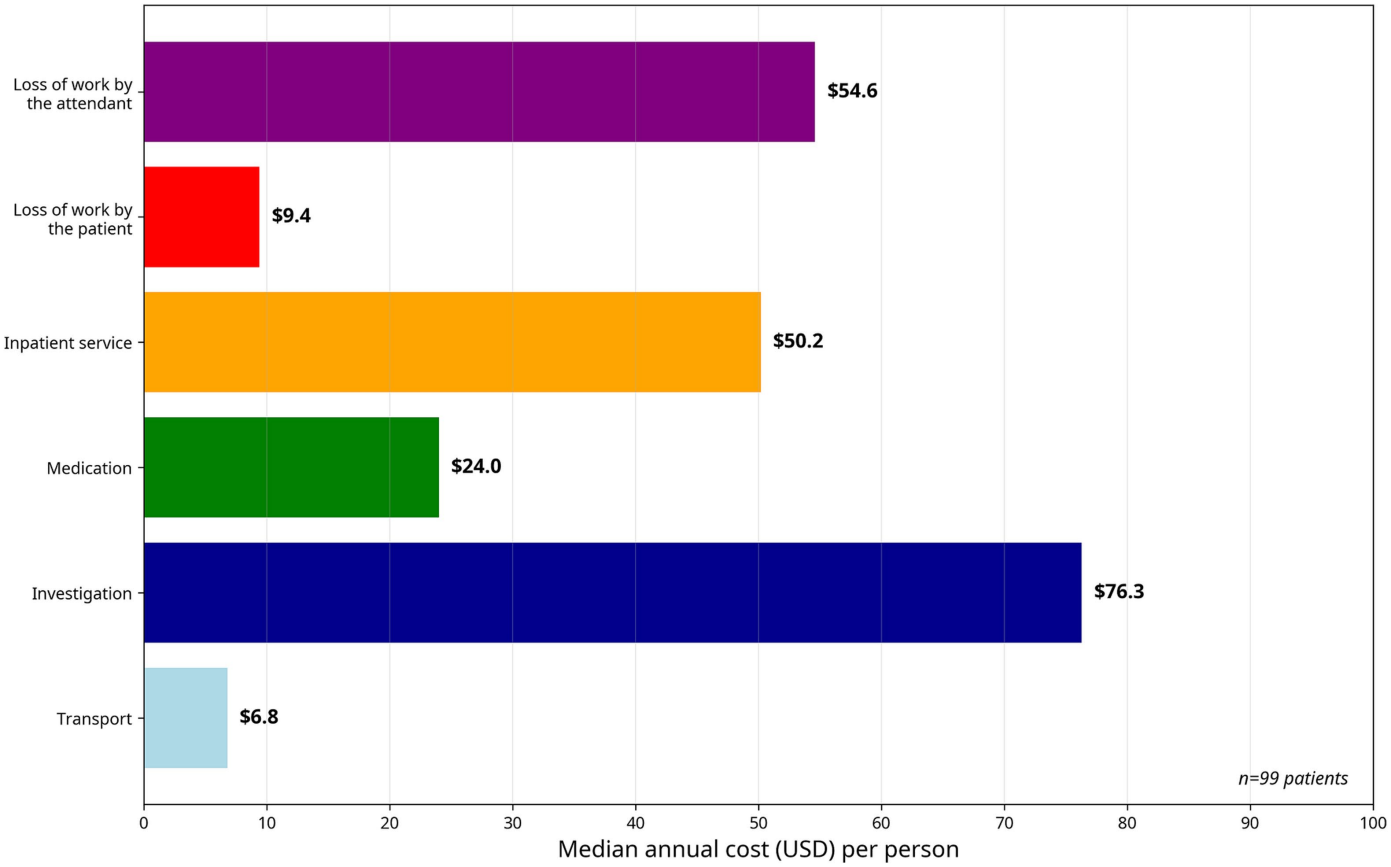
Cost of each component of acute stroke care in Ethiopian public hospitals.

In this study, indirect costs were considered the monetary value of lost productivity due to the absence of patients from work and their caregivers during the acute stroke care period, so total indirect costs were the sum of indirect patient costs and indirect caregiver costs. The median indirect patient cost was $9.41 and that of the caregivers was $54.6. Total acute stroke care costs were the sum of direct and indirect stroke care costs. Accordingly, the median cost of acute stroke care in these hospitals was $286 per patient during their course of care in the hospital. Figure 1 demonstrates the components of the cost of acute stroke care. Based on stroke type, it was $361.37 for ischemic stroke and $263.67 for hemorrhagic stroke per patient during their course of care in the hospital (Table 3).

**Table 3:** Cost of acute stroke care, in USD, among patients admitted to Tikur Anbessa Specialized Hospital and Yekatit 12 Hospital Medical College, Addis Ababa, 2021 (*n* = 99).

List of costs^a^	Minimum	Maximum	Median	IQR
Direct costs				
Transport cost	0	390	6.77	0–18.1
Investigation cost	4.29	243.3	76.26	58.6–89.2
Medication cost	2.84	594.6	23.98	13.9–45.7
Inpatient service cost	4.51	169.2	50.16	22.3–66.9
Other costs	0	383.6	0	0–11.3
Total direct cost	55.7	844.2	193.77	124.5–268.5
Indirect costs				
Patients cost^b^	0	180.5	9.41	0–33.8
Attendants cost	0	406.1	54.6	20.3–124.1
Total indirect cost	0	406.1	74	37–148.9
Total cost	71.48	847	286	162.3–439.3

a During the data collection period, the average exchange rate was 44.30 Ethiopian birr per 1 USD, and all monetary values were recorded in Ethiopian birr.

b Includes the monetary value of lost productivity due to the absence of patients from work during the acute stroke care period.

IQR, interquartile range; USD: United States Dollar.

### Factors associated with total acute stroke care cost

Higher between-group costs were independently associated with delayed hospital presentation, ICU admission, advanced age, comorbid conditions, and rural residence. Patients who arrived at the hospital more than 4.5 h after symptom onset had substantially higher expenditures than those presenting earlier, with mean total costs increased by about 78% (adjusted cost ratio [CR] 1.78, 95% CI: 1.35–2.35), highlighting delayed presentation as a major driver of resource use.

Admission to the ICU was also associated with higher expenditure (CR 1.56, 95% CI: 1.00–2.42; *p* = 0.049), consistent with the greater intensity and cost of intensive care. Older age contributed to increased costs: patients aged >60 years incurred approximately 27% higher mean costs than those aged ≤60 years (CR 1.27, 95% CI: 1.06–1.53). Similarly, having at least one comorbid condition was associated with a 27% higher mean cost compared with no comorbidity (CR 1.27, 95% CI: 1.03–1.56). Additionally, rural residence was associated with higher costs: rural residents had 40% higher mean expenses than urban patients (CR 1.40, 95% CI: 1.14–1.73), highlighting the extra economic burden on rural households (Table 4).

**Table 4:** Variables retained after bivariate screening in the multivariable analysis of total hospitalization costs among acute stroke patients admitted to Tikur Anbessa Specialized Hospital and Yekatit 12 Hospital Medical College, Addis Ababa, 2021 (*n* = 99)^a^.

Characteristics	Geometric mean cost^b^ (USD)	Cost ratio (95% CI)	*p*-value^c^
Age (in years)			
≤60	276 ± 46	Reference	0.013*
>60	351 ± 51	1.27 (1.06–1.53)	
Marital status			
Not married	288 ± 48	Reference	0.108
Married	337 ± 49	1.17 (0.97–1.42)	
Residence			
Urban	263 ± 36	Reference	0.002*
Rural	369 ± 67	1.40 (1.14–1.73)	
Type of stroke			
Hemorrhagic	284 ± 44	Reference	0.066
Ischemic	341 ± 54	1.20 (0.99–1.45)	
Comorbidity			
Absent	276 ± 47	Reference	0.026*
Present	351 ± 51	1.27 (1.03–1.56)	
Onset to treatment (hours)			
≤4.5	233 ± 45	Reference	<0.001*
>4.5	416 ± 57	1.78 (1.35–2.35)	
Admission			
General ward	249 ± 23	Reference	0.040*
ICU	389 ± 100	1.56 (1.002, 2.42)	
Medical complications			
No	286 ± 44	Reference	0.106
Yes	339 ± 56	1.19 (0.96–1.47)	

a A Cox–Snell-type pseudo-*R*^2^ = 47.38% (overall model performance).

b Values are presented as adjusted geometric means (± standard error) estimated using a generalized linear model with Gamma distribution and log link.

c Two-sided *p*-values are based on Wald *χ*^2^ tests; p* < 0.05 is considered statistically significant.

Deterministic sensitivity analyses showed that total cost estimates were robust across plausible variations in key cost drivers, with length of hospital stay and ICU admission remaining the main contributors to cost variability (Supplementary Table 3; Supplementary Figure 1).

## Discussion

Improving acute stroke care can reduce long-term disability and have a beneficial impact on the economic burden associated with stroke. Cost studies help understand the economic impact of stroke and allow decision-makers to understand the cost of stroke and better allocate healthcare resources. To our knowledge, this study is the first to systematically assess the cost of acute stroke in Ethiopia. In this study, the cost of acute stroke care was variable and strongly correlated with the length of hospital stay and ICU admission, both of which may be related to stroke severity.

The median cost of acute stroke care per patient per year was $286, with direct and indirect costs of $193.77 and $74, respectively. Direct costs represented approximately 67.8% of the total cost of acute care, which is very close to a previously done Indian study, which was 65% (7). The reported cost of acute stroke care per admission in China was $903 (current inflation-adjusted cost of $911.84) (8); $1,179 in Pakistan [current inflation-adjusted cost of $1,255.6 (9)], $4,984 in Singapore (current inflation-adjusted cost of $5,033.84) (10); and $6,887 in Japan (same as current cost) (11).

A breakdown of direct and indirect costs of acute stroke care in our study shows that the main cost elements are investigation costs, with a median cost of $76.26 (26.7%) of total costs, followed by attendants’ indirect costs of $54.6 (19.1%). Inpatient service costs of $50.16 (17.5%) and medication costs of 23.98 (8.4%) were the third and fourth major components of acute stroke care costs, respectively. These cost components differ from previous international studies where ward cost was the major component in Thailand, Singapore, and Japan with 57.6, 38.2, and 69%, respectively (10–12).

The highest investigation cost in our study can be explained by the fact that all the study hospitals did not have complete imaging and laboratory investigations; as a result, a significant number of patients were sent to private diagnostic centers and laboratories to complete their investigations. According to a 2014 review of African studies published by the World Stroke Foundation, the cost of direct stroke care in Nigerian government hospitals was $1,043 (currently $1,126.44, adjusted for inflation) and in Senegal was $416 (currently $430.68, adjusted for inflation) (4). Our result is $193.77 direct costs of stroke care, which is lower than the lowest results previously reported in Senegal ($430.68).

According to the World Bank, the GDP per capita of Ethiopia and Senegal in 2021 was $923.1 and $1,636.9, respectively (13). When we compare the two results in terms of GDP per capita, 21% of income in this study and 26.3% in Senegal go to direct stroke treatment costs. The median length of hospital stays was 9 days. The median daily acute stroke cost was obtained by dividing the median total acute stroke care cost by the median length of hospital stay, resulting in $21.53 per day. The length of hospital stay and the ICU admission are the strongest acute stroke care cost-determining factor in this study. These findings were supported by deterministic sensitivity analyses, which demonstrated that total acute care costs were most sensitive to utilization-related factors, particularly length of hospital stay and ICU admission, while overall cost estimates remained robust across plausible alternative assumptions (Supplementary Table 1; Supplementary Figure 1). In the multivariable analysis, delayed hospital presentation, ICU admission, advanced age, comorbid conditions, and rural residence appeared to be the strongest predictors of increasing acute stroke care costs.

When we compare the duration of hospitalization with the previously reported average value of seven countries (Pakistan, Brazil, India, Thailand, China, Turkey, and Malaysia), it is somehow similar, which was about nine and a half days (4). Current stroke costs can be reduced by shortening hospital stays. This can be achieved by putting in place early support for discharge and rehabilitation at home.

ICU admission due to stroke severity or the presence of other medical complications or comorbidities associated with increased costs of acute stroke care. In addition, intensive care medical service costs are higher than hospital ward service costs. All these factors explain the increase in the cost of admissions to intensive care units. The significant effect of rural residence on acute stroke care costs can be explained by transportation costs, and as often as not, people in rural areas tend to have more than one attendant, which directly increases indirect costs. Another explanation is that they have to go through multiple referrals from medical institutions at all levels to reach tertiary hospitals, which directly increases the direct cost.

Because thrombolytic therapy had not been initiated nationwide at the time of this study, stroke type had no significant effect on the total cost of acute stroke care. This result is consistent with previous studies in India and Malaysia (4). Interestingly, the average cost of ischemic stroke care ($361.4) was higher than that of hemorrhagic stroke ($263.7). This may be partly explained by the exclusion of severe strokes requiring mechanical ventilation from the analysis, which are mostly hemorrhagic strokes patients. As a result, the exclusion of more severe cases may have led to an underestimation of costs in the hemorrhagic group, making the average cost appear higher in ischemic stroke cases. Even though the costs of acute stroke care in public tertiary hospitals in Ethiopia are slightly lower than previously conducted African studies, this amount of money is very high for the majority of Ethiopians, where the average monthly salary is $186.59.^1^^,^^2^

The study has limitations that should be considered when interpreting the findings. The relatively small, sample of 99 patients from two urban tertiary hospitals, with exclusion of very severe cases requiring mechanical ventilation and those with major comorbid conditions, limits generalizability and likely underestimates the true economic burden of stroke. Although exclusion of mechanically ventilated patients may have resulted in underestimation of costs for severe stroke, sensitivity analyses using proxy high-cost scenarios suggest that this limitation is unlikely to materially affect the overall conclusions regarding the main cost drivers. The analysis is restricted to a 12-month horizon and considers only the patient and family perspective, omitting longer-term costs and system-level or broader societal impacts, including permanent disability, long-term rehabilitation, and institutional care. Some cost and productivity estimates rely on patient and caregiver recall and the use of average unit prices when receipts or exact payments were unavailable, introducing potential recall and measurement bias. In addition, the absence of stroke units, limited in-house diagnostic capacity, reliance on private diagnostic centers, and the predominance of out-of-pocket payments make the cost structure highly context-specific and may not be directly applicable to settings with different service organization or financing. Finally, the observational cost-of-illness design permits only associative, not causal, inferences, and costs were not associated with functional outcomes or quality of life. Therefore, the study cannot assess the cost-effectiveness of specific interventions or care pathways.

This study has several strengths. To our knowledge, it is the first multicenter, incidence-based, prospective cost-of-illness analysis of acute stroke care in Ethiopia, conducted at two large tertiary referral hospitals that manage a high volume of stroke patients. Using CT/MRI stroke diagnoses, NIHSS scoring at admission, and a pretested interviewer-led questionnaire enabled systematic collection of sociodemographic, clinical, and economic data. The micro-costing approach, applied prospectively from index admission through 12 months of follow-up, captured both direct and indirect patient-level costs, providing granular estimates of the financial burden of acute stroke care. In addition, appropriate regression methods for skewed cost data identified independent cost drivers, strengthening the relevance of the findings for health system planning and policy in low-income settings.

Organizing acute stroke care by implementing effective and cost-effective strategies, such as stroke units and reperfusion therapy, is essential to reduce the impact of the disease (14). Stroke units and intravenous thrombolysis are not only cost-effective but also save public health costs after the second year of implementation by improving functional outcomes, increasing the chance of returning to work, reducing the length of hospital stay, decreasing the likelihood of complications, reducing the need for ICU care, and lowering the risk of readmission (14, 15). Despite this, a recent study conducted by the World Stroke Organization evaluating the status of stroke center implementation worldwide (16), demonstrated that the region with the fewest stroke units is sub-Saharan Africa. Understanding the cost of stroke in Ethiopia is essential for planning the implementation of acute care services, including stroke treatments within universal health coverage, so that it can truly benefit all stroke patients in the country.

## Data Availability

The raw data supporting the conclusions of this article will be made available by the authors, without undue reservation.

## Footnotes

1 HelloPebl. Average salary in Ethiopia. Available online at: https://hellopebl.com/resources/blog/average-salary-in-ethiopia/ (Accessed March 22, 2026).

2 Paylab. Salary comparison in Ethiopia. Available online at: https://www.paylab.com/et/salaires-dans-le-pays?global_currency=USD (Accessed May 22, 2026).
