# Supplementary material for: Assessing the cost of acute stroke care in Ethiopian public tertiary hospitals: a multicenter study
Source: Front Neurol. 2026 Mar 27;17:1664986. doi: 10.3389/fneur.2026.1664986 (PMC13065655; doi:10.3389/fneur.2026.1664986)
Supplement: Supplementary file 1 [file Table_1.DOCX]

**SUPPLEMENTARY MATERIAL**

**Costing and unit-cost estimation**

**Supplementary Table 1. Unit costs applied in the micro-costing of investigations in Ethiopian birr (ETB) in 2021.**

| **Dataset variable** | **Cost item / service** | **Unit (per…)** | **Mean unit cost used in analysis (ETB)** | **SD (range across private-sector providers) (ETB)** |
| --- | --- | --- | --- | --- |
| CT SCAN | Non-contrast brain CT scan | Scan | 1575 | 724 (1,500–4000) |
| MRI | Brain MRI | Scan | 4160 | 1049 (3000–6000) |
| CT and MRI | CT + MRI for same episode | Episode with both modalities | 2074 | 1534 (4500–8500) |
| CBC | Complete blood count | Test | 142 | 142 (300–700) |
| Coag profile | Coagulation profile | Panel | 259 | 364 (400–1500) |
| LFT | Liver function tests | Panel | 195 | 114 (300–600) |
| RFT | Renal function tests | Panel | 126 | 82 (100–400) |
| Electrolyte | Serum electrolytes | Panel | 499 | 445 (1000–2250) |
| CRP | C-reactive protein | Test | 8 | 33 (70–160) |
| ESR | Erythrocyte sedimentation rate | Test | 15 | 18 (50–80) |
| lipid profile | Lipid profile | Panel | 160 | 116 (100–400) |
| FBS | Fasting blood sugar | Test | 16 | 18 (50–60) |
| viral markers | Viral markers (HBV/HCV/HIV etc.) | Panel | 29 | 104 (250–721) |
| Urine analysis | Urine analysis | Test | 27 | 51 (150–400) |
| Cardiac biomarker | Cardiac biomarkers | Panel | 12 | 39 (100–186) |
| ECG | Electrocardiogram | Test | 16 | 24 (50–200) |
| Echocardiography | Echocardiography | Test | 71 | 96 (300–800) |
| Chestxry | Chest X-ray | Film | 41 | 44 (100–160) |
| others | Other investigations (e.g., carotid Doppler, etc.) | Test / procedure | 127 | 131 (500–1010) |

N.B: All costs are expressed in 2021 ETB. For investigations performed in private-sector facilities, the table reports the mean unit cost and the standard deviation or minimum–maximum range across providers.

**Computation of individual-level cost variables**

Individual-level direct and indirect cost variables were constructed from the raw dataset using a standardized, patient-level micro-costing approach. For each patient, quantities of resource use were multiplied by the relevant unit costs from Table 1 and then summed across cost categories. Table 2 describes the definition and computation of each cost variable used in the analysis.

**Supplementary Table 2. Definition and computation of individual-level cost variables.**

| Cost variable | How it was computed at patient level (step-by-step) | Variables used |
| --- | --- | --- |
| DCTransport | Total amount (ETB) paid for all transport related to the index stroke episode (referral facilities and study hospitals), as reported by the patient or caregiver. No unit-cost assumptions were applied. | DCTransport |
| DCInvestigation | Sum of costs of all investigations during acute care. For each patient, each investigation indicator (CTSCAN, MRI, CTandMRI, coagprofile, CBC, LFT, RFT, Electrlyte, CRP, ESR, lipidprofile, FBS, viralmarkers, Urineanalysis, Cardiacbiomarker, ECG, Echocardiography, Chestxry, others) was multiplied by the corresponding unit cost from Table 1 and then summed. Formally: DCInvestigationᵢ = Σₖ Iᵢₖ × UCₖ. | CTSCAN, MRI, CTandMRI, coagprofile, CBC, LFT, RFT, Electrlyte, CRP, ESR, lipidprofile, FBS, viralmarkers, Urineanalysis, Cardiacbiomarker, ECG, Echocardiography, Chestxry, others |
| DCMedication | Total expenditure on prescribed stroke-related medicines and consumables during admission, based on hospital pharmacy bills and, when purchased externally, average prices from private pharmacies. | DCMedication |
| DCServices | Total inpatient service cost (bed, nursing care, professional fees) during the index admission, taken directly from hospital billing records for the medical ward and ICU. | DCServices, Duration, Site |
| DCOther | Other direct medical and non-medical costs related to the current stroke (e.g., other facility visits before reaching the study hospitals) as reported by patients or caregivers. | DCOther |
| DCTotal | Total direct cost of acute stroke care per patient. Computed as: DCTotalᵢ = DCTransportᵢ + DCInvestigationᵢ + DCMedicationᵢ + DCServicesᵢ + DCOtherᵢ. | DCTransport, DCInvestigation, DCMedication, DCServices, DCOther |
| IDCPatient | Productivity loss of the patient during the acute admission, valued using the human capital approach: number of days off work multiplied by the patient’s average daily income. | IDCPatient (derived from days off work and income) |
| IDCAttendants | Productivity loss of all attendants during the acute admission: sum of days off work for each attendant multiplied by his or her average daily income. | IDCAttendants |
| IDCTotal | Total indirect cost per patient. Computed as: IDCTotalᵢ = IDCPatientᵢ + IDCAttendantsᵢ. | IDCPatient, IDCAttendants |
| SumTotalCost | Overall cost of acute stroke care per patient (societal perspective), used as the main dependent variable in the cost-determinants analysis. Computed as: SumTotalCostᵢ = DCTotalᵢ + IDCTotalᵢ. | DCTotal, IDCTotal |

| Parameter varied | Base-case assumption | Lower-bound scenario | Upper-bound scenario | Resulting total cost (USD) |
| --- | --- | --- | --- | --- |
| Length of hospital stay | 9 days | 6 days  (25th percentile) | 12 days  (75th percentile) | ~US$ 170 – ~US$ 380 |
| Investigation costs | Median  (US$ 76.3) | 25th percentile  (US$ 58.8) | 75th percentile (US$ 89.5) | ~US$ 260 – ~US$ 320 |
| Inpatient service costs | Median | −20% | +20% | ~US$ 255 – ~US$ 320 |
| Medication costs | Median | −25% | +25% | ~US$ 275 – ~US$ 300 |
| Indirect costs (caregivers) | Median  (US$ 74) | −30% | +50% | ~US$ 265 – ~US$ 330 |
| Transportation costs | Median | Urban-level costs | Rural-level costs | ~US$ 270 – ~US$ 310 |
| ICU admission | No ICU | — | ICU admission | ~US$ 500 – ~US$ 550 |
| In-hospital complications | None | — | ≥1 complication | ~US$ 410 – ~US$ 450 |
| Severe cases excluded (proxy) | Excluded | — | +10–15% high-cost cases | ~US$ 320 – ~US$ 360 |

**Supplementary Table 3. Deterministic Sensitivity Analyses of Total Acute Stroke Care Cost per Patient**

Base-case (median): US$ 286 per patient (12,679 birrs)

**Sensitivity analyses** were conducted to assess the robustness of total acute stroke care cost estimates to uncertainty in key cost drivers and structural assumptions. Deterministic one-way sensitivity analyses varied major parameters individually, including length of hospital stay, investigation expenses, inpatient service costs, medication costs, transportation fees, caregiver-related indirect costs, ICU admission, and in-hospital complications. Parameter ranges were established based on the empirically observed 25th and 75th percentiles, as well as minimum–maximum values or clinically plausible bounds derived from the study data.

Scenario analyses were performed to incorporate combined variations in key parameters across low-cost, base-case, and high-cost scenarios. Furthermore, a conservative scenario was developed to account for the exclusion of mechanically ventilated patients by using higher cost assumptions to proxy for groups characterized by ICU admission, severe neurological deficits, or in-hospital complications.

Across all analyses, estimated total costs were most sensitive to utilization-related factors, particularly **length of hospital stay, ICU admission, and diagnostic testing**. In contrast, variation in medication costs and indirect productivity losses had a smaller relative effect on overall estimates. These findings indicate that the base-case results are robust and highlight the potential for cost reduction through improved organization of acute stroke care, early supported discharge, and expansion of stroke unit–based services.

**Supplementary Figure 1. Tornado Plot of One-Way Sensitivity Analyses for Total Acute Stroke Care Cost**


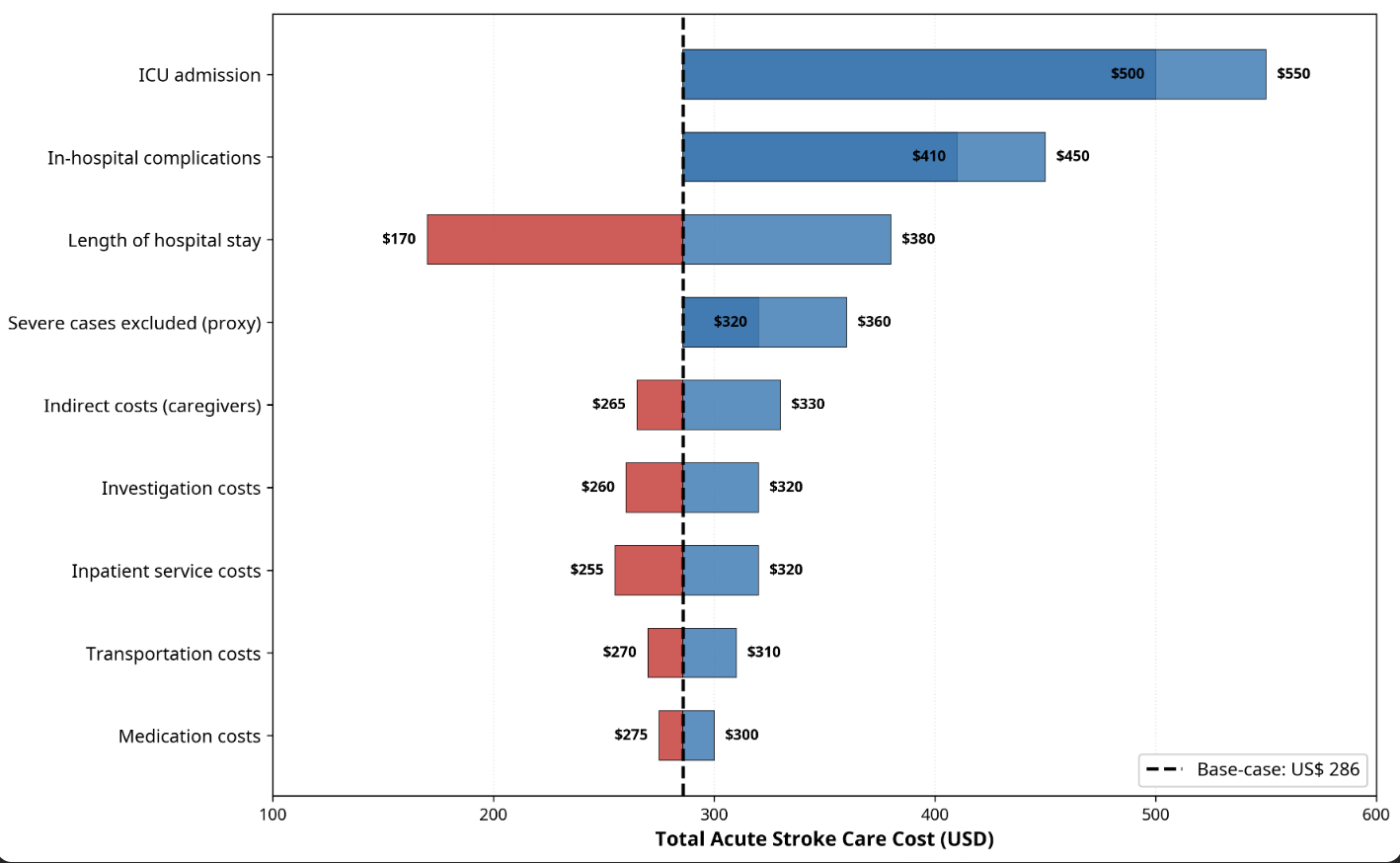


Supplementary Figure 1 presents a Tornado Plot showing the results of one-way deterministic sensitivity analyses of the total acute stroke care cost in Ethiopia. The base-case cost estimate was US$ 286 per patient (median of 12,679 birrs). The Tornado Plot shows how changes in individual cost parameters affect the total cost of acute stroke care when each parameter is varied independently between its lower-bound (25th percentile) and upper-bound (75th percentile) scenarios, while all other parameters remain fixed at their base-case values.

The horizontal bars in the plot extend from the base-case cost (marked by a dashed vertical line at US$ 286) in both directions, indicating the range of total costs resulting from parameter variations. Bars extending to the left (shown in red) indicate scenarios with cost reductions, while bars extending to the right (shown in blue) indicate scenarios with increased costs. Parameters are arranged vertically in ascending order of their maximum impact on total cost, making it easy to identify the most influential cost drivers.

A one-way deterministic tornado plot was created to rank parameters based on their impact on the median total cost of acute stroke care. The widest bars represented the length of hospital stay, followed by ICU admission, investigation costs, and in-hospital complications. Parameters concerning medication expenses and indirect productivity losses exhibited narrower ranges and contributed less significantly to overall cost estimates. Overall, utilization-related and structural factors had a greater influence on total costs than unit-price variation.
