# Supplementary figures and images for: Assessing the cost of acute stroke care in Ethiopian public tertiary hospitals: a multicenter study
Source: Front Neurol. 2026 Mar 27;17:1664986. doi: 10.3389/fneur.2026.1664986 (PMC13065655; doi:10.3389/fneur.2026.1664986)

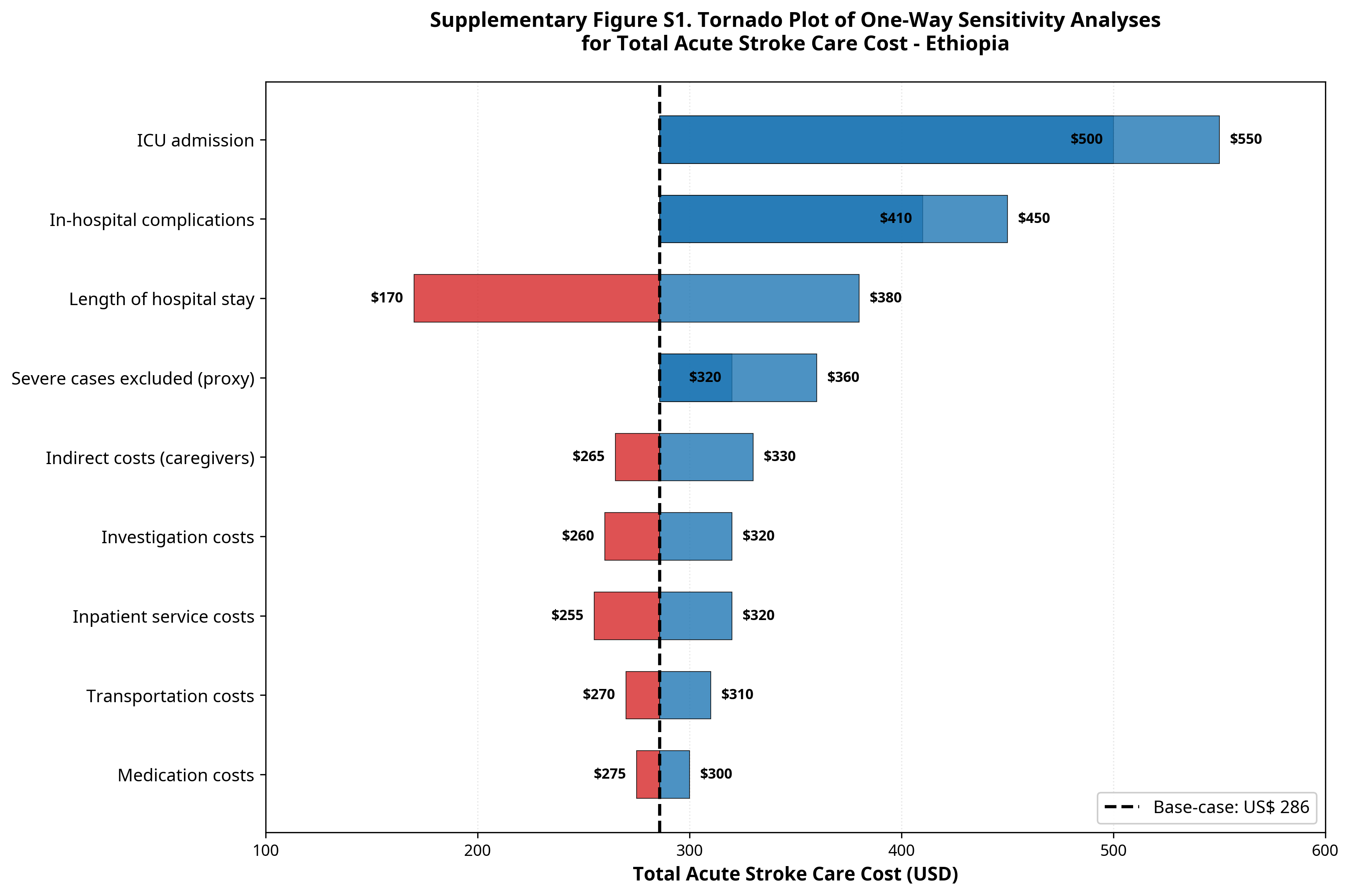

Supplement: Supplementary file 2 [file Image_1.PNG]
